# Cardiac Self‐Efficacy Mediates the Relationship Between Patient Empowerment and Medication Adherence: A Path Analysis in Patients With Coronary Artery Disease ‐ Empirical Research Quantitative

**DOI:** 10.1002/nop2.70837

**Published:** 2026-10-04

**Authors:** Atefeh Allahbakhshian, Fatemeh Hashempour, Samin Saiyadani, Parvin Sarbakhsh, Masood Mohammadnezhad, Fatemeh Radimaher

**Affiliations:** ^1^ Department of Medical Surgical Nursing Tabriz University of Medical Sciences Tabriz Iran; ^2^ Student Research Committee Tabriz University of Medical Sciences Tabriz Iran; ^3^ Department of Statistic and Epidemiology Tabriz University of Medical Sciences Tabriz Iran; ^4^ Education and Life Sciences Birmingham City University Birmingham UK

**Keywords:** cardiac self‐efficacy, coronary artery disease, mediation, medication adherence, path analysis, patient empowerment, self‐determination

## Abstract

**Aim:**

To examine the mediating role of cardiac self‐efficacy in the relationship between patient empowerment and medication adherence among patients with coronary artery disease (CAD) and to identify which empowerment sub‐dimensions are most prominent.

**Design:**

A cross‐sectional correlational study.

**Methods:**

A convenience sample of adults attending a cardiology clinic completed validated versions of the Coronary Artery Disease Empowerment Scale (CADES), Cardiac Self‐Efficacy Scale and Morisky Medication Adherence Scale. Data were analysed using Spearman's correlations, ordinal logistic regression and path analysis with bootstrap mediation testing.

**Results:**

Positive correlations were found among empowerment, self‐efficacy and adherence. Regression analysis showed that cardiac self‐efficacy predicted adherence, while empowerment did not directly predict adherence. Path analysis confirmed that self‐efficacy mediates the empowerment‐adherence relationship. The self‐determination subscale of empowerment was the strongest predictor of self‐efficacy, whereas personal competence was not significant.

**Conclusion:**

Patient empowerment enhances medication adherence indirectly by building cardiac self‐efficacy, primarily through fostering patient autonomy rather than merely providing knowledge.

**Implications for the Profession and/or Patient Care:**

Nurse‐led strategies should target cardiac self‐efficacy. Motivational interviewing and autonomy‐supportive interventions may be more effective than traditional education.

**Impact:**

- What problem did the study address? The study addressed the gap in understanding the nurse‐led interventions that translate patient empowerment into actual medication adherence behaviours.
- What were the main findings? Self‐efficacy acts as the essential bridge between empowerment and adherence and this bridge is built most effectively through self‐determination rather than personal competence.
- Where and on whom will the research have an impact? These results will impact clinical nurses, nurse educators and healthcare professionals involved in cardiac care by highlighting that nurse‐led counselling strategies and autonomy‐supportive interventions may be more effective for improving medication.

**Reporting Method:**

We adhered to the STROBE guidelines.

**Patient or Public Contribution:**

No Patient or Public Contribution.

## 1 Introduction

Effective management of coronary artery disease (CAD) is fundamentally dependent on patient self‐management, with steadfast medication adherence being a cornerstone of secondary prevention (Chen et al. 2022). Non‐adherence is a pervasive clinical problem that compromises therapeutic goals, increases morbidity and raises healthcare costs (Kleinsinger 2018; Wan et al. 2022; Yang et al. 2025). This persistent gap between prescribed therapies and patient behaviour signals that traditional, didactic patient education is often insufficient. Consequently, healthcare providers are increasingly focused on patient‐centred counselling strategies that foster the internal psychological resources required for sustained self‐care (Perveen et al. 2025; Xu et al. 2025).

Within this patient‐centred framework, two constructs are paramount: patient empowerment and self‐efficacy. Patient empowerment is a process through which individuals gain the knowledge, skills and, crucially, the confidence to take an active, autonomous role in their health decisions (Jalali et al. 2024; Kim et al. 2021; Zhang et al. 2024). This concept is distinct from, yet theoretically linked to, self‐efficacy, which is defined within Social Cognitive Theory (SCT), the guiding framework for this study, as an individual's specific belief in their capability to execute the behaviours necessary to manage their condition, such as taking medications correctly even when faced with challenges (Bandura 2001; Xiong et al. 2024).

## 2 Background

A robust body of evidence confirms the independent value of these constructs. Patient empowerment is increasingly recognized as a positive correlate of self‐efficacy across chronic conditions (Acuña Mora et al. 2022; Aujoulat et al. 2008; Náfrádi et al. 2017; Wang et al. 2022). Empowerment‐based interventions, which often include goal‐setting and shared decision‐making, have been shown to improve psychological resources and, in some cases, long‐term cardiovascular health outcomes (Hajialibeigloo et al. 2021; Lu et al. 2024; Petersson et al. 2022; Wang et al. 2022). Separately, self‐efficacy is consistently identified as one of the most powerful and proximal predictors of medication adherence and other self‐care behaviours (Gruszczyńska et al. 2021; Kalantzi et al. 2023; Náfrádi et al. 2017). While the literature supports that empowerment and self‐efficacy are both beneficial, a critical gap exists in understanding how they work together. It is theoretically plausible that empowerment does not influence behaviour directly, but rather that its primary role is to build self‐efficacy, which in turn drives adherence. This mediation pathway (Empowerment ➔ Self‐Efficacy ➔ Adherence) has been established for other antecedents, such as health literacy, but it has not been empirically validated for the empowerment‐adherence relationship in CAD patients (Huang et al. 2018).

Furthermore, empowerment itself is a complex, multidimensional concept. A key debate in the literature questions whether empowerment is merely the paternalistic helping process of transferring knowledge and skills (i.e., personal competence) or a deeper redistribution of power that strengthens a patient's self‐determination and autonomy (Halvorsen et al. 2020). It is unknown which of these specific dimensions of empowerment is most critical for building a patient's self‐efficacy. Finally, these psychological models are predominantly studied in Western populations; their applicability within different sociocultural contexts, such as a Middle Eastern population, remains largely unexplored.

Therefore, the present study was designed to address these gaps by testing a hypothesized mediation model in a sample of Middle Eastern patients with CAD.

## 3 The Study

### 3.1 Aim(s) and Objective

Our primary aim was to determine if the relationship between patient empowerment and medication adherence is mediated by cardiac self‐efficacy. A secondary aim was to identify which specific sub‐dimensions of empowerment (e.g., self‐determination, personal competence) are the most salient predictors of self‐efficacy.

Based on the theoretical framework, we hypothesized the following:

*Patient empowerment will be positively associated with medication adherence*.

*Patient empowerment will be positively correlated with cardiac self‐efficacy*.

*Cardiac self‐efficacy will be positively correlated with medication adherence*.

*Cardiac self‐efficacy will mediate the relationship between patient empowerment and medication adherence*.

By elucidating this specific psychological pathway, this research aims to provide an empirical basis for designing more targeted and effective counselling strategies. If self‐efficacy is indeed the essential bridge, clinical practice must shift from simply informing patients to actively building their belief in themselves.

## 4 Methods/Methodology

### 4.1 Design

This study employed a cross‐sectional correlational design to examine the relationships between empowerment, self‐efficacy and medication adherence among patients with CAD. This design allowed for the assessment of associations between variables as they naturally occur, without experimental manipulation. This study is reported in accordance with the Strengthening the Reporting of Observational Studies in Epidemiology (STROBE) guidelines for cross‐sectional studies (File S1).

### 4.2 Instrument With Validity and Reliability

A validated questionnaire was used to collect data, comprising four components as follows: Demographic and Clinical Characteristics: This section was used to collect demographic and clinical information, including age, gender, marital status, educational level, occupation, income, living situation, duration of disease and family history of CAD. All these variables were considered to be independent variables.

Coronary Artery Disease Empowerment Scale (CADES): Patient empowerment was measured using the 25‐item CADES, developed by Kim et al. (2021). This scale assesses patients' perceived control and active role in managing their heart disease. It includes three subscales: Self‐determination (12 items), Emotional self‐regulation (7 items) and Personal competence (6 items). Items are rated on a 5‐point Likert scale, yielding a total score range of 25 to 125, where higher scores indicate greater levels of empowerment. The Farsi version of the CADES, validated by Jalali et al. (2024) has demonstrated acceptable psychometric properties, including good internal consistency (Cronbach's *α* = 0.813) (Jalali et al. 2024). In the conceptual model, patient empowerment served as the independent variable.

Sullivan's Cardiac Self‐Efficacy Scale (SCSES): Self‐efficacy was assessed with the 13‐item SCSES, developed by Sullivan et al. (1998). This instrument measures a patient's confidence in their ability to manage CAD, broken down into two subscales: maintaining function (5 items) and symptom control (8 items). Items are rated on a 5‐point Likert scale, yielding a total score range of 13 to 65, where higher scores indicate greater levels of self‐efficacy. Varaei et al. (2017) validated the Farsi version of SCSES, demonstrating excellent reliability with a Cronbach's alpha of 0.977 (Varaei et al. 2017). Self‐efficacy served as the proposed mediating variable in the model.

Morisky Medication Adherence Scale (MMAS‐8): Medication adherence was measured using the 8‐item Morisky Medication Adherence Scale (Morisky 2008). This widely used self‐report tool assesses specific adherence behaviours and barriers (e.g., Do you ever forget to take your medicine?). This scale utilizes a mixed scoring system (seven dichotomous items and one 5‐point Likert item), yielding a total score ranging from 0 to 8, with scores classified as high adherence (8), medium adherence (6–7), or low adherence (< 6). Iranpour et al. (2022) validated the Persian version of the 8‐item MMAS‐8, demonstrating acceptable reliability with a Cronbach's alpha of 0.70 (Iranpour et al. 2022). Medication adherence served as the dependent (outcome) variable.

### 4.3 Sampling and Recruitment

A convenience sampling method was employed. Data were collected from patients referred to the Cardiology Clinic over the course of the year 2025. This specific, university‐affiliated clinic was selected as the sole study site because it functions as a major referral center for cardiology patients from across the Northwest region of the country, thus providing a large and accessible sample representative of the target population. Trained research assistants approached individuals in the clinic waiting area and screened them for eligibility.

### 4.4 Sample Size and Power

A priori power analysis using G*Power 3.1 indicated that a minimum sample of 259 participants was required to detect a small‐to‐medium effect size (*f* ^2^ = 0.05) for the regression models with two predictors, given an alpha of 0.05% and 90% power. We aimed to recruit 300 participants to account for potential incomplete data. The final sample of 266 represents the total number of participants who provided complete responses to all three primary scales.

### 4.5 Population and Sample

The study population consisted of adult patients with a confirmed diagnosis of CAD who were attending a university‐affiliated cardiology clinic for routine follow‐up. All participants were in a non‐acute phase of their illness. The routine follow‐up criterion included any scheduled, non‐acute visit, regardless of whether it was the patient's first attendance at the clinic.

### 4.6 Inclusion and/or Exclusion Criteria

The study population consisted of patients meeting the following criteria: (1) age 18 years or older, (2) a confirmed diagnosis of CAD, (3) attending the clinic for a routine follow‐up, (4) ability to provide informed consent and (5) a current prescription for at least one daily oral medication for CAD. While no specific minimum duration for the CAD diagnosis was set, the exclusion of recent acute events ensured all participants were in a non‐acute phase. Patients were excluded if they had documented cognitive impairments or had experienced an acute cardiac event (e.g., myocardial infarction) within the 30 days preceding data collection.

### 4.7 Data Sources/Collection

Following informed consent, research assistants provided standardized verbal instructions to participants on how to complete the self‐report questionnaires. For illiterate patients, a structured interview was conducted where questions were read verbatim. All questionnaires were checked for completeness on‐site before the participant left.

### 4.8 Data Analysis

All data were analysed using IBM SPSS Statistics, Version 28 and IBM SPSS AMOS, Version 28. A *p*‐value of < 0.05 was considered statistically significant. Descriptive statistics (frequencies, percentages, means, standard deviations (SD) and medians with interquartile ranges (IQR) for medication adherence) were calculated to summarize participant characteristics and the main study variables.

Given the ordinal and mixed‐response structure of the Medication Adherence Scale (MMAS‐8), non‐parametric methods were applied for analyses involving medication adherence as the outcome variable. Associations between medication adherence and categorical sociodemographic and clinical characteristics were examined using the Mann–Whitney *U* test for two‐group comparisons and the Kruskal–Wallis test for variables with more than two categories. Preliminary relationships among empowerment, cardiac self‐efficacy and medication adherence were examined using Spearman's rank‐order correlation coefficient.

To examine the independent predictors of medication adherence, an ordinal logistic regression model (proportional odds model) was employed. The proportional odds assumption was tested and satisfied. The model included patient empowerment and cardiac self‐efficacy as predictors.

Furthermore, path analysis with observed (manifest) variables rather than latent variable structural equation modelling (SEM) was employed for the following reasons: (a) the primary aim was to examine the structural relationships between dimensions of empowerment, cardiac self‐efficacy and medication adherence, rather than to validate the measurement instruments; (b) all measures used in this study have established psychometric properties with demonstrated reliability (Cronbach's α ranging from 0.79 to 0.92), allowing the use of total and subscale scores; and (c) sample size considerations favoured a more parsimonious model to ensure stable parameter estimates and to avoid overparameterization (Kline 2016). Using total scores (or subscale scores) is a form of parcelling, which is a commonly accepted practice in SEM when the sample size is limited relative to the number of indicators (Little et al. 2002). Parcelling reduces model complexity, improves the ratio of sample size to estimated parameters and increases the stability of parameter estimates (Bandalos 2002). However, we acknowledge that using observed scores means measurement error is not explicitly modelled (Cole and Preacher 2014), which represents a limitation of our approach that we address in the Discussion section.

To test the hypothesized theoretical model and the mediating role of self‐efficacy (Hypothesis H_4_), a path analysis was conducted using AMOS 28. The path model specified empowerment subscales as exogenous variables, cardiac self‐efficacy subscales as mediators and medication adherence as the outcome. Consistent with the hypothesized model, no direct paths from empowerment subscales to medication adherence were included. The primary model was estimated using maximum likelihood (ML) estimation, treating the MMAS‐8 total score as an observed composite score. To address concerns regarding the ordinal nature of medication adherence, a Bayesian estimation approach was additionally performed in AMOS using Markov Chain Monte Carlo (MCMC) estimation. The Bayesian model demonstrated comparable parameter estimates with the original model; therefore, the primary results are presented in the main manuscript, while Bayesian estimates are provided as File S2.

Model fit was assessed using established indices: the chi‐square to degrees of freedom ratio (*χ* ^2^/df), the Comparative Fit Index (CFI), the Tucker‐Lewis Index (TLI) and the Root Mean Square Error of Approximation (RMSEA) with its 90% confidence interval (CI). Acceptable model fit was considered when *χ* ^2^/df < 3.0, CFI and TLI > 0.95 and RMSEA < 0.06. The posterior predictive *p*‐value (PPP) and the Deviance Information Criterion (DIC) were examined as indicators of Bayesian model fit. Standardized direct and indirect effects were calculated to evaluate the hypothesized mediation pathways.

To assess the statistical significance of the indirect (mediation) effects, we employed a bootstrap resampling approach (Preacher and Hayes 2008). Bias‐corrected (BC) 95% confidence intervals were computed based on 1000 bootstrap samples. An indirect effect was considered statistically significant if the 95% BC confidence interval did not contain zero. This approach does not assume normality of the sampling distribution of the indirect effects and provides more robust inference for mediation analysis.

### 4.9 Ethical Considerations

Ethical approval was obtained from the institutional review board and the study complied with the ethical principles outlined in the Declaration of Helsinki.

## 5 Results

### 5.1 Subjects

During the recruitment period, a total of 352 patients attending the clinic were approached and screened for eligibility. Of these, 52 were excluded (19 did not meet the inclusion criteria, 33 declined to participate). A total of 300 patients consented and received the questionnaires. The final sample for analysis consisted of 266 adult participants who provided complete responses to all primary scales, yielding a completion rate of 88%. The mean age of the sample was 53.56 years (SD = 13.57), with ages ranging from 18 to 86. The majority of participants were female (52.6%, *n* = 140), married (79.7%, *n* = 212) and reported an income less than their expenses (61.7%, *n* = 164). Regarding their initial diagnosis, 76.4% of participants had a history of acute coronary syndrome (ACS), while 23.6% presented with stable, non‐ACS CAD. Regarding clinical characteristics, 67.7% had hypertension, 22.2% had diabetes and 44.7% had hyperlipidemia. Participants were prescribed a mean of 4.12 (SD = 1.45) daily cardiovascular medications. The most frequently prescribed medication classes included antiplatelets (91.4%), lipid‐lowering agents (84.2%) and antihypertensives (72.6%). Most participants (43.2%) reported a disease duration of more than 4 years. Descriptive statistics for the key study variables are presented in Table 1.

**TABLE 1:** Sociodemographic and clinical characteristics and their association with empowerment, medication adherence and self‐efficacy scores.

Characteristics	Group/category	*N* (%)	Empowerment (Mean ± SD)	Self‐determination (Mean ± SD)	Emotional self‐regulation (Mean ± SD)	Personal competence (Mean ± SD)	Medication adherence, median (Q1–Q3)	Cardiac self‐efficacy (Mean ± SD)	Symptom control (Mean ± SD)	Maintaining function (Mean ± SD)
Gender ^t^	Female	140 (52.6%)	98.29 ± 13.71	46.42 ± 7.96	28.84 ± 4.42	23.04 ± 3.80	6 (4.5–7.75)	27.29 ± 10.60	9.87 ± 4.71	17.41 ± 6.88
	Male	126 (47.4%)	97.06 ± 13.72	46.21 ± 8.28	28.58 ± 4.47	22.42 ± 4.03	6.5 (4.5–7.5)	29.26 ± 11.11	10.85 ± 4.84	18.41 ± 7.28
	*p*		0.476	0.835	0.593	0.276	0.729	0.208	**0.043**	0.284
Marital status ^a^	Single	29 (10.9%)	99.72 ± 11.34	46.55 ± 7.02	29.83 ± 3.99	23.34 ± 3.85	6 (3.75–7)	30.52 ± 11.08	11.03 ± 5.05	19.48 ± 7.09
	Married	211 (79.6%)	97.85 ± 13.83	46.54 ± 8.21	28.70 ± 4.48	22.69 ± 3.93	6.13 (4.5–7.75)	28.47 ± 10.53	10.37 ± 4.75	18.10 ± 6.81
	Divorced	8 (3.0%)	100.38 ± 12.36	46.75 ± 8.46	29.25 ± 3.20	24.38 ± 1.41	6.75 (6.375–7.875)	31.88 ± 14.33	11.50 ± 6.48	20.38 ± 8.38
	Widowed	17 (6.4%)	91.35 ± 15.46	42.94 ± 8.19	26.76 ± 4.78	21.65 ± 4.46	5.5 (3.5–6.75)	19.47 ± 9.47	8.18 ± 3.63	11.29 ± 6.67
	*p*		0.203	0.368	0.156	0.328	0.178	**0.004**	0.208	**< 0.001**
Patient education ^a^	Illiterate	57 (21.5%)	91.61 ± 14.74	42.91 ± 8.94	27.30 ± 4.64	21.40 ± 4.01	5.75 (3.5–7)	22.05 ± 9.57	8.47 ± 4.43	13.58 ± 6.22
	Below diploma	86 (32.5%)	95.69 ± 12.86	45.41 ± 7.77	28.17 ± 4.56	22.10 ± 3.97	6.5 (4.5–7.75)	27.36 ± 9.59	9.95 ± 4.40	17.41 ± 6.44
	Diploma	72 (27.2%)	100.04 ± 12.49	47.40 ± 7.01	29.11 ± 4.17	23.53 ± 3.58	5.75 (3.75–6.875)	28.71 ± 9.52	10.03 ± 4.33	18.68 ± 6.27
	Bachelor's	38 (14.3%)	106.13 ± 8.94	50.29 ± 6.02	31.18 ± 2.62	24.66 ± 3.45	7 (5.5–7.75)	36.29 ± 10.44	13.53 ± 5.07	22.76 ± 6.29
	***p***		**< 0.001**	**< 0.001**	**0.001**	**0.001**	**0.026**	**< 0.001**	**< 0.001**	**< 0.001**
Occupation ^a^	Housewife	109 (41.1%)	96.80 ± 14.03	46.03 ± 8.21	28.39 ± 4.62	22.38 ± 3.89	5.75 (4.5–7)	25.39 ± 9.57	9.06 ± 4.26	16.33 ± 6.40
	Employee	34 (12.8%)	106.53 ± 11.08	50.49 ± 7.93	31.29 ± 2.80	25.06 ± 2.84	7.75 (5.375–8)	39.71 ± 10.35	14.94 ± 4.74	24.77 ± 6.54
	Self‐employed	59 (22.3%)	97.86 ± 11.66	46.75 ± 6.52	28.25 ± 4.33	22.86 ± 4.12	6.75 (3.5–7.375)	27.75 ± 9.44	9.78 ± 4.55	17.97 ± 5.89
	***p***		**0.002**	**0.016**	**0.008**	**0.004**	**0.065**	**< 0.001**	**< 0.001**	**< 0.001**
Income ^a^	Less than expenses	164 (61.9%)	95.42 ± 14.54	44.62 ± 8.60	28.30 ± 4.66	22.50 ± 3.90	5.75 (3.75–7)	25.51 ± 10.15	9.40 ± 4.32	16.12 ± 6.81
	Equal to expenses	90 (34.0%)	101.39 ± 11.43	49.05 ± 6.41	29.29 ± 4.06	23.20 ± 3.81	6.75 (4.75–7.75)	32.49 ± 10.60	11.99 ± 4.90	20.51 ± 6.73
	***p***		**0.002**	**< 0.001**	0.138	0.395	0.064	**< 0.001**	**< 0.001**	**< 0.001**
Disease duration ^a^	6 Months— 1 Year	76 (28.7%)	99.66 ± 11.20	47.84 ± 6.82	29.21 ± 4.31	22.80 ± 3.84	6.75 (5–7.75)	29.95 ± 12.32	10.43 ± 5.35	19.52 ± 7.76
	1 to 2 Years	33 (12.5%)	102.85 ± 10.83	48.73 ± 6.19	30.00 ± 3.14	24.12 ± 3.78	6.75 (5.5–7.75)	32.12 ± 11.62	12.55 ± 5.03	19.58 ± 6.86
	2 to 4 Years	41 (15.5%)	100.05 ± 11.18	47.27 ± 7.07	29.32 ± 3.22	23.46 ± 2.98	6.5 (4.5–7.75)	28.80 ± 9.17	11.05 ± 3.69	17.76 ± 5.92
	> 4 Years	115 (43.4%)	94.12 ± 15.82	44.27 ± 9.24	27.80 ± 5.04	22.05 ± 4.18	5.5 (3.75–6.75)	25.74 ± 9.65	9.38 ± 4.46	16.36 ± 6.76
	***p***		**0.001**	**< 0.001**	**0.025**	**0.011**	**0.002**	**0.002**	**< 0.001**	**0.011**

*Note:* The table presents associations between sociodemographic/clinical characteristics and scores of empowerment, cardiac self‐efficacy, and medication adherence. Continuous psychological variables (patient empowerment, self‐efficacy, and its subscales) are presented as mean ± standard deviation (SD) and were compared using independent samples *t*‐tests (indicated by ^t^) or one‐way analysis of variance (ANOVA) (indicated by ^a^). Medication adherence, measured by the MMAS‐8, is presented as median (Q1–Q3) due to its ordinal/non‐normal distribution and was compared using non‐parametric tests (Mann–Whitney *U* test for two‐group comparisons and Kruskal–Wallis test for comparisons involving more than two groups). The reported *p*‐values correspond to the appropriate statistical tests for each variable. Statistically significant *p*‐values are indicated in bold (*p* < 0.05). Percentages may not sum to 100% due to rounding. Exact *p*‐values are reported in the respective column.

Participants reported a mean total empowerment score of 97.71 (SD = 13.06) and a mean cardiac self‐efficacy score of 50.12 (SD = 9.92). The mean medication adherence score on the MMAS‐8 was 6.0 (IQR 4.5–7.75), indicating a medium level of adherence on average.

Internal consistency for the scales was assessed using Cronbach's alpha. All scales demonstrated good to excellent internal consistency in this sample: The 25‐item CADES demonstrated excellent reliability (*α* = 0.87). The MMAS‐8 showed acceptable reliability (*α* = 0.75). The 13‐item SCSES demonstrated excellent reliability (*α* = 0.92).

Although the distributions of empowerment and medication adherence deviated from normality according to the Shapiro–Wilk test (*p*s < 0.05), medication adherence was primarily analysed using rank‐based non‐parametric methods because of the ordinal nature of the MMAS‐8. The distribution for cardiac self‐efficacy was approximately normal (*r*_s_ = 0.990, *p* = 0.057).

Spearman's rank‐order correlation was conducted to examine the relationships among the key variables. As hypothesized, all three variables were positively and significantly correlated. There was a statistically significant, positive correlation between medication adherence and patient empowerment, *r*_s_ (264) = 0.24, *p* < 0.001. A statistically significant, positive correlation was also found between medication adherence and cardiac self‐efficacy, *r*_s_ (264) = 0.44, *p* < 0.001. Finally, a statistically significant, positive correlation was observed between total patient empowerment and cardiac self‐efficacy, *r*_s_ (264) = 0.49, *p* < 0.001. Table 2 summarizes the Spearman's correlation coefficients among the study measures.

**TABLE 2:** Spearman's Rho (*ρ*) correlations among medication adherence, empowerment, and cardiac self‐efficacy.

Variable	Medication adherence	Em‐ powerment	Cardiac self‐efficacy
Medication adherence	—		
Empowerment	**0.238**	—	
Cardiac self‐efficacy	**0.442**	**0.487**	—

*Note:* N = 266. Correlation coefficients are Spearman's rho. Bold values indicate statistical significance at the *α* < 0.05 level (two‐tailed). Exact *p*‐values for all correlations were < 0.001 and are reported in the text.

The ordinal logistic regression model was statistically significant (*χ* ^2^ = 69.16, df = 2, *p* < 0.001), indicating that the set of predictors significantly improved the model fit compared to the intercept‐only model. As shown in Table 3, cardiac self‐efficacy was a significant positive predictor of higher medication adherence (*B* = 0.086, SE = 0.012, Wald = 48.30, *p* < 0.001; OR = 1.09, 95% CI [1.06, 1.12]). This indicates that a one‐unit increase in cardiac self‐efficacy score was associated with a 9% increase in the odds of being in a higher adherence category. However, total patient empowerment was not a significant predictor in this model (*B* = 0.011, SE = 0.009, Wald = 1.51, *p* = 0.219; OR = 1.01, 95% CI [0.99, 1.03]). The pseudo *R* ^2^ values indicated that the model explained approximately 23.1% of the variability in adherence levels according to Nagelkerke's pseudo *R* ^2^.

**TABLE 3:** Ordinal logistic regression predicting medication adherence (*N* = 266).

Variable	*B* (SE)	Wald	df	*p*	OR (95% CI)
Empowerment	0.011 (0.009)	1.508	1	0.219	1.01 (0.99–1.03)
Cardiac Self‐Efficacy (CSES)	0.086 (0.012)	48.302	1	**< 0.001**	1.09 (1.06–1.12)

*Note:* Statistically significant *p*‐values are indicated in bold (*p* < 0.05). Exact *p*‐values are reported in the *p*‐value column. The proportional odds assumption was satisfied test of parallel lines: *χ* ^2^ = 69.16, df = 2, *p* < 0.001.

Abbreviations: CI, confidence interval; OR, odds ratio.

Before conducting the path analysis, the assumptions of multivariate normality and the absence of multivariate outliers were evaluated, as maximum likelihood estimation is sensitive to violations of these assumptions (Kline 2016). Univariate normality was assessed using skewness and kurtosis values. As shown in Table 4, all variables exhibited acceptable skewness (ranging from −0.765 to 0.099) and kurtosis (ranging from −0.747 to 0.293), well within the recommended thresholds (skewness < |3.0|, kurtosis < |8.0|; Kline 2016). Multivariate normality was assessed using Mardia's coefficient of multivariate kurtosis (6.032, c.r. = 5.021), which, while slightly elevated, is within acceptable limits for path analysis with an adequate sample size. Multivariate outliers were identified using Mahalanobis distance (d‐squared). The critical value for *χ* ^2^ with 6 degrees of freedom at *p* < 0.001 is 22.46 (Tabachnick and Fidell 2013). Only four observations (1.8% of the sample) exceeded this threshold. Given the low percentage of potential outliers (< 5%), all cases were retained in the analysis, as excluding them would not substantially affect the results (Tabachnick and Fidell 2013). Overall, the data met the assumptions for path analysis using maximum likelihood estimation.

**TABLE 4:** Assessment of univariate and multivariate normality for study variables.

Variable	Skewness	Critical ratio	Kurtosis	Critical ratio
Empowerment‐ Personal Competence	−0.354	−2.360	−0.587	−1.954
Empowerment‐ Emotional Self‐Regulation	**−0.711**	**−4.731**	0.293	0.976
Empowerment‐ Self‐Determination	**−0.678**	**−4.517**	0.078	0.259
Cardiac Self‐Efficacy‐ Cardiac Self‐Efficacy	0.099	0.661	−0.495	−1.648
Cardiac Self‐Efficacy‐ Maintaining Function	−0.082	−0.546	−0.747	−2.487
Medication Adherence	**−0.765**	**−5.093**	−0.266	−0.886
Multivariate			**6.032**	**5.021**

*Note:* Critical ratios greater than ±1.96 indicate significant deviation from normality at *p* < 0.05. Statistically significant critical ratios are indicated in bold. Multivariate kurtosis = 6.032, c.r. = 5.021.

The path analysis model specified patient empowerment (with sub‐factors self‐determination, emotional self‐regulation and personal competence) as the exogenous variable predicting self‐efficacy (with sub‐factors symptom control and maintaining function), which in turn predicted the final outcome, medication adherence (see Figure 1).

**FIGURE 1:**
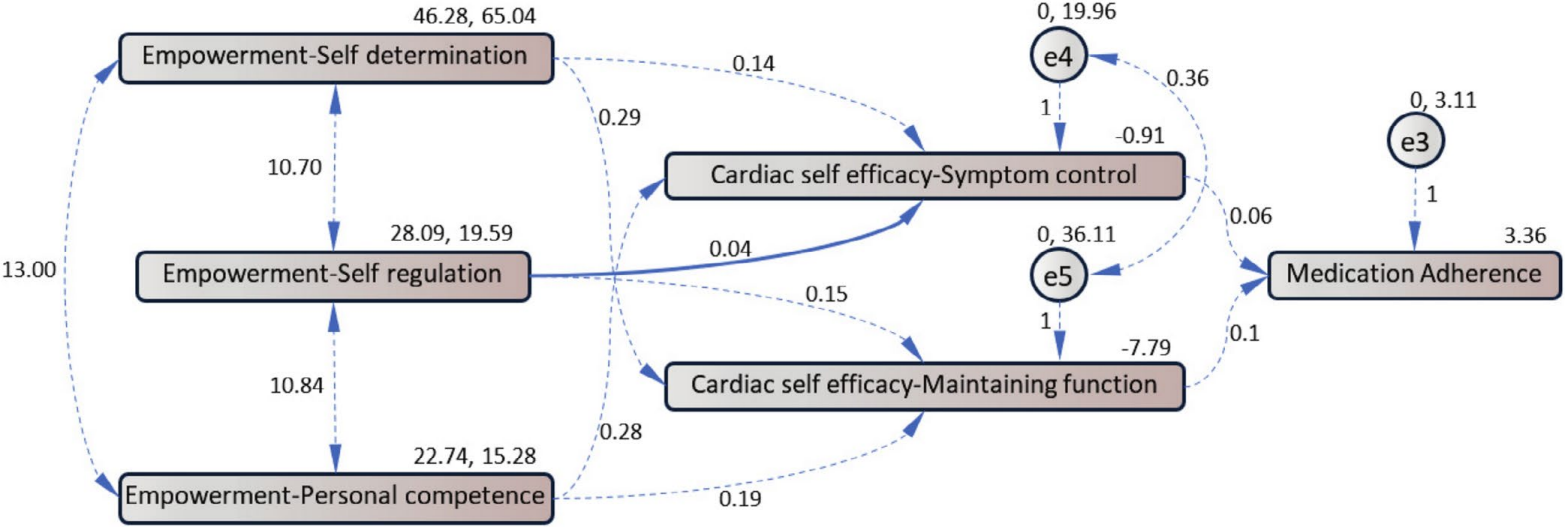
Standardized path model of cardiac self‐efficacy mediating the relationship between patient empowerment subscales and medication adherence (*N* = 266). Note: Standardized path coefficients for the hypothesized full mediation model. Solid lines represent statistically significant paths (*p* < 0.05), whereas dashed lines represent non‐significant paths (*p* ≥ 0.05). The model demonstrated excellent fit indices: χ^2^/df = 1.354, CFI = 0.998, TLI = 0.990, RMSEA = 0.037.

#### 5.1.1 Step 1: Empowerment ➔ Self‐Efficacy

The analysis revealed that the self‐determination subscale of empowerment was a significant positive predictor of both self‐efficacy subscales: ‘symptom control’ (*β* = 0.25, *p* < 0.001) and ‘maintaining function’ (*β* = 0.33, *p* < 0.001). The emotional self‐regulation subscale also positively predicted ‘maintaining function’ (*β* = 0.17, *p* = 0.019).

Notably, the personal competence subscale of empowerment was not a significant predictor of either ‘symptom control’ (*β* = 0.12, *p* = 0.109) or ‘maintaining function’ (*β* = 0.10, *p* = 0.125).

#### 5.1.2 Step 2: Self‐Efficacy ➔ Medication Adherence

As hypothesized, cardiac self‐efficacy strongly and significantly predicted medication adherence. Both ‘maintaining function’ (*β* = 0.34, *p* < 0.001) and ‘symptom control’ (*β* = 0.15, *p* = 0.038) had significant positive paths to medication adherence.

To formally test the hypothesized mediation effects and to obtain robust confidence intervals, we calculated the indirect effects of each empowerment subscale on medication adherence through the self‐efficacy subscales using bias‐corrected bootstrap with 1000 resamples. Table 5 presents the direct, indirect and total effects with their 95% BC bootstrap confidence intervals.

**TABLE 5:** Direct and indirect effects of empowerment and cardiac self‐efficacy components on medication adherence in the mediation model with bias‐corrected 95% bootstrap confidence intervals (1000 samples).

Independent variable	Dependent variable	Path	Effect type	Estimate	SE	bc 95% lower	BC 95% upper	*p*	Mediation status
Empowerment	Cardiac self‐efficacy	Self‐Determination → symptom control	Direct	0.150	0.041	0.067	0.231	< 0.001	***
		Self‐determination → Maintaining function	Direct	0.298	0.055	0.182	0.402	< 0.001	***
		Emotional self‐regulation → Maintaining function	Direct	0.304	0.117	0.094	0.523	0.009	**
		Emotional self‐regulation → Symptom control	Direct	0.066	0.087	−0.091	0.235	0.449	Not significant
		Personal competence → Maintaining function	Direct	0.142	0.120	−0.063	0.335	0.236	Not significant
		Personal competence → Symptom control	Direct	0.108	0.089	−0.065	0.251	0.227	Not significant
Empowerment	Medication Adherence	Personal competence → Medication adherence	Indirect	0.020	0.016	−0.010	0.053	0.211	Not significant
		Emotional self‐regulation → Medication adherence	Indirect	0.033	0.018	0.002	0.072	**0.042**	Significant
		Self‐determination → Medication adherence	Indirect	0.038	0.010	0.020	0.061	**0.002**	Significant
Cardiac Self‐Efficacy	Medication Adherence	Maintaining function → Medication adherence	Direct	0.095	0.021	0.058	0.141	**0.002**	—
		Symptom control → Medication adherence	Direct	0.063	0.031	0.000	0.120	0.050	—

*Note:* Statistically significant values are indicated in bold (*p* < 0.05). Based on 1000 bootstrap samples.

Abbreviations: BC, Bias‐Corrected; CI, Confidence Interval; SE, Standard Error.

^**^ *p* 〈 0.01.

^***^ *p* 〈 0.001.

The indirect effect of Self‐determination on medication adherence was significant (estimate = 0.038, BC 95% CI [0.020, 0.061], *p* = 0.002), indicating that self‐determination positively influences adherence through enhanced cardiac self‐efficacy. Similarly, the indirect effect of Emotional self‐regulation was significant (estimate = 0.033, BC 95% CI [0.002, 0.072], *p* = 0.042). In contrast, the indirect effect of Personal competence was not significant (estimate = 0.020, BC 95% CI [−0.010, 0.053], *p* = 0.211), as the confidence interval crossed zero.

Regarding the direct effects of the self‐efficacy subscales on adherence, maintaining function showed a strong direct effect (estimate = 0.095, bc 95% CI [0.058, 0.141], *p* = 0.002) and symptom control demonstrated a borderline significant direct effect (estimate = 0.063, bc 95% CI [0.000, 0.120], *p* = 0.050).

The hypothesized model specified no direct paths from the empowerment subscales to medication adherence; instead, the empowerment subscales were linked to adherence only through cardiac self‐efficacy. Therefore, the total effects for the empowerment subscales equal their indirect effects. The significant indirect effects for self‐determination and emotional self‐regulation support the hypothesized mediation pathway, while the non‐significant indirect effect for personal competence suggests that this dimension does not operate through cardiac self‐efficacy. Because direct paths from empowerment to adherence were not estimated in the specified model, these findings should be interpreted as evidence for an indirect‐only mediation model rather than as a formal statistical proof of full mediation. These bootstrap results provide robust evidence that self‐determination and emotional self‐regulation are the key empowering dimensions that drive adherence through the building of self‐efficacy.

To evaluate the robustness of the findings given the ordinal nature of the MMAS‐8, Bayesian estimation was additionally performed using Markov Chain Monte Carlo (MCMC) estimation in AMOS.

Convergence Diagnostics: MCMC convergence was assessed using standard diagnostics. The Potential Scale Reduction Factor (PSRF/R‐hat) was 1.000 for all parameters, well below the recommended threshold of 1.10 (Gelman and Rubin 1992), indicating adequate convergence. Trace plots visually confirmed chain stationarity with no trends. Additionally, the posterior predictive *p*‐value (PPP = 0.44) indicated excellent model fit and the Deviance Information Criterion (DIC = 52.44) with an effective number of parameters (pD = 23.69) was computed.

The Bayesian model demonstrated highly comparable parameter estimates and substantive conclusions to those obtained using the maximum likelihood approach. Therefore, the maximum likelihood results are presented in the main manuscript, whereas the full details of Bayesian diagnostics, including trace plots, are provided in File S3.

## 6 Discussion

This study's primary contribution is the empirical validation of a mediation model where the effect of patient empowerment on medication adherence in CAD patients is fully channelled through cardiac self‐efficacy. While our initial analysis confirmed positive correlations between empowerment, self‐efficacy and adherence, our path analysis revealed a more sophisticated relationship. Because the final model specified no direct path from empowerment to adherence, the observed association was modelled as operating through self‐efficacy; the indirect path through self‐efficacy was strong and significant. This finding provides an empirical pathway that supports conceptual models used in major systematic reviews, suggesting that self‐efficacy is the more proximal and direct driver of adherence (Gruszczyńska et al. 2021; Náfrádi et al. 2017). This aligns with its foundational role in SCT and with other research identifying self‐efficacy as a key translational mechanism; for instance, it has also been found to mediate the relationship between health literacy (i.e., numeracy) and medication adherence (Bandura 2001). This positions self‐efficacy as a central, critical mechanism that translates various patient assets into behavioural action (Huang et al. 2018).

The strong, direct relationship found between cardiac self‐efficacy and medication adherence confirms our third hypothesis (H_3_) and aligns with a vast body of literature (Náfrádi et al. 2017; Wan et al. 2022). Both the regression analysis and the mediation model demonstrated that cardiac self‐efficacy was a significant predictor of medication adherence. This finding is consistent with systematic reviews which have concluded that high levels of self‐efficacy are consistently found to promote medication adherence across various chronic conditions (Náfrádi et al. 2017). Our findings confirm this principle holds true for this specific population of Middle Eastern CAD patients. Furthermore, a recent review of patient‐related factors influencing adherence in cardiometabolic patients confirmed that psychosocial constructs, including self‐concept and patient beliefs (of which self‐efficacy is a core component), are a primary category of factors determining adherence to overall treatment (Kalantzi et al. 2023).

If self‐efficacy is the key, this study's findings also clarify how to build it, confirming our second hypothesis (H_2_) that empowerment is a critical antecedent. This was evidenced by the strong, positive correlation between the two constructs (*r*_s_ = 0.49) and the significant paths from empowerment subscales to self‐efficacy subscales. This link is robustly supported in the literature; a recent descriptive review of empowerment correlates found that self‐efficacy is one of the most frequently identified and significant correlates of patient empowerment (Acuña Mora et al. 2022). This cross‐sectional model is strongly supported by intervention studies. Empowerment‐based interventions have been shown to significantly improve not only self‐efficacy but also closely related psychological resources such as resilience in patients recovering from coronary artery stent implantation (Hajialibeigloo et al. 2021; Petersson et al. 2022; M. Wang et al. 2021). For instance, a randomized controlled trial (RCT) by Moattari et al. (2012) found that an empowerment program significantly improved self‐efficacy, including decision‐making, in haemodialysis patients (Moattari et al. 2012). Similarly, an RCT with liver transplant patients demonstrated that empowerment education, based on Self‐Determination Theory, significantly enhanced self‐efficacy (Guo et al. 2023). Our data provide a theoretical model that helps explain why such empowerment interventions are successful: they build the necessary psychological reserves, like self‐efficacy, that are required for sustained self‐care.

A particularly salient finding was the contrast between the empowerment subscales: Self‐determination was the strongest predictor of both self‐efficacy factors, while personal competence had no significant effect. The non‐significant path from personal competence suggests that merely providing patients with knowledge and skills (i.e., education) is insufficient to build the robust confidence required for daily disease management. This finding challenges models that equate empowerment with education and supports the more complex critique by Aujoulat et al. (2008), who argue that true empowerment is not just about gaining control or skills, but also involves finding meaning and coherence (Aujoulat et al. 2008).

Conversely, the strong path from self‐determination empirically demonstrates a core critique within empowerment theory. Halvorsen et al. (2020), in a synthesis of empowerment concept analyses, argued that empowerment is often misconstrued as a paternalistic helping process of making patients act differently (Halvorsen et al. 2020). They argue that true empowerment is not just about gaining skills but is a redistribution of power characterized by mutual partnership, reciprocity, trust… and a process of strengthening self‐determination (Halvorsen et al. 2020). Our data provide tangible evidence for this conceptual distinction: the intervention that merely provides personal competence (the helping model) failed to build self‐efficacy, whereas the factor related to autonomy and Self‐determination (the power redistribution model) was the significant driver. This aligns perfectly with the Self‐Determination Theory (SDT) framework, which posits that supporting patient autonomy is the key to internalising motivation and enhancing self‐efficacy (Ryan and Deci 2000).

The bootstrap analysis provided robust support for the mediation model. The significant indirect effects of self‐determination (BC 95% CI [0.020, 0.061]) and emotional self‐regulation (BC 95% CI [0.002, 0.072]) through cardiac self‐efficacy, combined with the non‐significant direct effects of empowerment subscales on adherence, confirm that self‐efficacy fully mediates this relationship. The use of bias‐corrected bootstrap confidence intervals strengthens our confidence in this conclusion, as this method does not rely on the assumption of normality for the indirect effect distribution. Importantly, the non‐significant indirect effect of personal competence (BC 95% CI [−0.010, 0.053]) further underscores that merely providing knowledge and skills is insufficient; rather, fostering autonomy and emotional regulation is crucial for building the self‐efficacy that translates into adherence behaviour.

Our finding of a non‐significant direct path from empowerment to adherence in the mediation model (failing to support H_1_ in the final model) may seem to contradict other studies, but it actually provides a more sophisticated explanation. While empowerment and adherence were significantly correlated in our initial analysis (*r*_s_ = 0.24), this relationship vanished once self‐efficacy was introduced as a mediator. Other cross‐sectional (Náfrádi et al. 2017) and intervention studies (Macedo et al. 2017) have reported a direct, significant relationship between empowerment and adherence. Our findings suggest the relationship they observed is likely explained by self‐efficacy, a variable they did not concurrently model as a mediator. Therefore, our model does not refute their findings but rather provides the missing causal link, positioning self‐efficacy as the full mediator of this relationship.

The clear pathway identified in this study, from self‐determination to self‐efficacy to adherence, has direct implications for clinical practice and future research. To improve medication adherence, clinical interventions should not just aim to empower patients with information (which relates to our non‐significant Personal competence factor). Instead, they must specifically target and build cardiac self‐efficacy. Our model suggests the most effective way to do this is by enhancing a patient's sense of Self‐determination, such as by using motivational interviewing or collaborative goal‐setting. This supports the call for empowerment‐based interventions in recent systematic reviews (Abadi et al. 2020).

For clinical nurses, these findings highlight the importance of moving beyond information‐based education toward nurse‐led interventions that strengthen patients' autonomy, shared decision‐making and cardiac self‐efficacy. Nursing interventions incorporating motivational interviewing, collaborative goal‐setting and individualized self‐management support may represent practical approaches to enhance medication adherence among patients with CAD.

Furthermore, our findings highlight a critical methodological point. We found a strong correlation (*r*_s_ = 0.49) between empowerment and cardiac‐specific self‐efficacy. In contrast, Kärner Köhler et al. (2018) found only a weak correlation (*r*_s_ = 0.38) between empowerment and general self‐efficacy in their CAD patient sample (Kärner Köhler et al. 2018). The stronger association in our study underscores the necessity for future research to use disease‐specific, rather than general, self‐efficacy measures, which may be more sensitive to these clinically meaningful relationships.

### 6.1 Limitations

These conclusions must be interpreted within the context of the study's limitations. Primarily, the cross‐sectional design precludes definitive causal inference. The relationship between adherence and self‐efficacy is likely bidirectional; successful adherence may, in itself, reinforce a patient's self‐efficacy over time. Empowerment is also described as a process of personal transformation, which further implies a temporal dynamic that our study could not capture. Also, cross‐sectional mediation may overestimate or bias effects compared to longitudinal models. Future studies should employ prospective longitudinal designs or interventional approaches to confirm the mediational pathway and establish the true directionality of these relationships.

Second, this study relied exclusively on self‐report measures for both the psychological constructs and adherence behaviours. Furthermore, we did not collect objective clinical biomarkers (such as lipid profiles, HbA1c, or resting blood pressure measurements) to assess actual cardiovascular risk factor control. Future research should incorporate objective adherence measures, such as pharmacy refill data or electronic pill monitoring, as well as clinical biomarkers, to validate findings, reduce bias and determine whether higher self‐efficacy reliably translates into optimal disease control.

Third, we used total observed (summated) scores rather than latent variables in our path analysis. As a result, measurement error is not explicitly modelled in the structural relationships (Cole and Preacher 2014). This means that the estimated path coefficients may be attenuated (biased toward zero) due to unreliability in the measures and the true relationships may be stronger than those we have reported. However, we opted for this approach for several reasons: (a) all measures used are well‐established instruments with demonstrated psychometric properties in previous validation studies (Cronbach's *α* ranging from 0.79 to 0.92), (b) parcelling into total scores reduces model complexity and improves the ratio of sample size to estimated parameters and (c) with our sample size, a full latent variable model would have been overparameterized, potentially leading to convergence issues or unstable estimates (Kline 2016). Despite this limitation, the significant mediation effects we observed are likely robust, as they emerged despite the conservative bias introduced by measurement error. Future research should employ latent variable SEM with multiple indicators per construct to explicitly model and correct for measurement error.

Fourth, the convenience sample from a single university clinic may not be generalisable to all CAD patients. Participants in this specific setting may differ systematically from broader patient populations concerning their socioeconomic status, baseline health literacy, or overall engagement with healthcare services.

Fifth, our mediation model did not account for other potentially influential psychological or contextual factors, such as comorbid depression, the quality of patient‐provider communication, or varying levels of health literacy. Because these unmeasured variables could partially explain the observed relationships, the conclusion of full mediation by cardiac self‐efficacy should be interpreted with caution.

Finally, because the cultural specificity of empowerment constructs may influence how subdimensions like self‐determination operate, future research should explore these dynamics across more diverse, cross‐cultural samples. It is imperative to examine whether clinical interventions specifically targeting self‐determination reliably produce measurable, longitudinal improvements in both cardiac self‐efficacy and adherence.

### 6.2 Recommendations for Further Research

Based on the limitations of this study, several directions for future research are recommended. First, prospective longitudinal designs or interventional approaches are needed to confirm the mediational pathway and establish the true directionality of the observed relationships. Second, future studies should incorporate objective adherence measures, such as electronic pill monitoring, pharmacy refill data or therapeutic drug monitoring, alongside clinical biomarkers (e.g., lipid profiles, blood pressure) to validate findings and reduce self‐report bias. Third, latent variable SEM with multiple indicators per construct should be employed to explicitly model and correct for measurement error. Fourth, given the cultural specificity of empowerment constructs, cross‐cultural studies are warranted to explore how subdimensions like self‐determination operate across diverse populations. Finally, it is imperative to examine whether clinical interventions specifically targeting self‐determination reliably produce measurable, longitudinal improvements in both cardiac self‐efficacy and medication adherence.

## 7 Conclusion

Despite these limitations, this study provides a crucial empirical model of the psychological pathway from empowerment to adherence in this population. It moves beyond simple correlation to propose an ordered, functional pathway, providing an empirical model of how a patient's sense of empowerment is translated into action. This study empirically confirms that self‐efficacy is the essential bridge that empowerment must cross to become behaviour, highlighting the central role of nurses in developing autonomy‐supportive interventions that enhance patients' cardiac self‐efficacy.

## Author Contributions

**Atefeh Allahbakhshian:** supervision, validation, writing – review and editing, resources, funding acquisition. **Fatemeh Hashempour:** investigation, data curation. **Samin Saiyadani:** investigation, project administration. **Parvin Sarbakhsh:** formal analysis, software, visualization, validation. **Masood Mohammadnezhad:** methodology, writing – review and editing. **Fatemeh Radimaher:** conceptualization, methodology, formal analysis, writing – original draft.

## Funding

This work was supported by the Student Research Committee, Tabriz University of Medical Sciences. The authors wish to express their appreciation to the authorities of the Student Research Committee for their support.

## Disclosure

During the preparation of this work, the authors used Chatgpt from Open AI for assistance in refining the English language and grammar of this manuscript. The authors reviewed and edited the output as needed and take full responsibility for the content of the published article. The statistician on the author team is Pravin Sarbakhsh.

Statistical Declaration: There is a statistician on the author team and state which author: Parvin SARBAKHSH. The authors affirm that the statistical methods used were appropriate for the study design and data characteristics, and that statistical findings were correctly interpreted.

## Conflicts of Interest

The authors declare no conflicts of interest.

## Supporting information


**File S1:** STROBE Statement Checklist for Cross‐Sectional Studies.

**File S2:** Bayesian Path Analysis as a Sensitivity Analysis.
**Table S2:** Bayesian Parameter Estimates for the Path Analysis Mode.

**File S3:** Bayesian MCMC Convergence and Bootstrap Stability Assessment.
**Figure S1:** Diagnostic plots for the path from Self‐Determination to Symptom Control.
**Figure S2:** Diagnostic plots for the path from Emotional Self‐Regulation to Maintaining Function.
**Figure S3:** Diagnostic plots for the path from Personal Competence to Maintaining Function.
**Figure S4:** Diagnostic plots for the path from Personal Competence to Symptom Control.
**Figure S5:** Diagnostic plots for the path from Self‐Determination to Maintaining Function.
**Figure S6:** Diagnostic plots for the path from Emotional Self‐Regulation to Symptom Control.
**Figure S7:** Diagnostic plots for the path from Symptom Control to Medication Adherence.
**Figure S8:** Diagnostic plots for the path from Maintaining Function to Medication Adherence.

## Data Availability

The data that support the findings of this study are available from the corresponding author upon reasonable request.
