# Supplementary material for: Cardiac Self‐Efficacy Mediates the Relationship Between Patient Empowerment and Medication Adherence: A Path Analysis in Patients With Coronary Artery Disease ‐ Empirical Research Quantitative
Source: Nurs Open. 2026 Oct 4;13(10):e70837. doi: 10.1002/nop2.70837 (PMC13635616; doi:10.1002/nop2.70837)
Supplement: Supplementary file 1 [file NOP2-13-e70837-s001.docx]

**Supplementary File 3.** Bayesian MCMC Convergence and Bootstrap Stability Assessment

To evaluate both the convergence of the Markov Chain Monte Carlo (MCMC) algorithm and the stability of the parameter estimates, two complementary sets of diagnostic plots are presented for each direct path in the model.

**A) MCMC Convergence Diagnostics via Trace Plots**

Trace plots (Figures S1A–S8A) display the evolution of parameter estimates across MCMC iterations. The horizontal axis represents the iteration number, and the vertical axis represents the estimated value. The observed patterns exhibit a "fuzzy caterpillar" shape with rapid, dense fluctuations around a stable mean and no systematic upward or downward trends. This pattern, combined with a Potential Scale Reduction Factor (PSRF/R-hat), reported in Amos as the Convergence Statistic (C.S.), of 1.000 for all parameters, which is well below the commonly recommended thresholds of 1.002 and 1.1, confirms successful chain convergence and stationarity (Gelman & Rubin, 1992). Additionally, since Amos does not report the Effective Sample Size (ESS) in its default tabular output, the number of samples retained after the thinning process was used as a practical indicator of sample adequacy. A total of [70,000] operational independent samples were retained for the final analysis, which is more than sufficient to achieve stable and reliable estimates. Collectively, the numerical indices and diagnostic plots confirm the successful and reliable convergence of the MCMC algorithm and the adequacy of the sample size for Bayesian inference in this study.

**B) Bootstrap Distribution Plots for Parameter Stability**

Bootstrap distribution plots (Figures S1B–S8B) display the frequency distribution of parameter estimates obtained from 1,000 bootstrap resamples. The horizontal axis represents the bootstrap replication number, and the vertical axis represents the frequency of each estimated value. The distributions are approximately unimodal and centered around the point estimates obtained from maximum likelihood estimation, supporting the robustness and stability of the findings.

**Figure S1. Diagnostic plots for the path from Self-Determination to Symptom Control.**

**A	B**

- (A) Trace Plot: Confirms MCMC convergence with stable stationarity around the mean estimate (≈0.15).
- (B) Bootstrap Distribution Plot: Shows the distribution of bootstrap estimates centered around the mean (≈0.15), indicating estimate stability.

**Figure S2. Diagnostic plots for the path from Emotional Self-Regulation to Maintaining Function.**

**A	B**

- (A) Trace Plot: Confirms MCMC convergence with stable stationarity around the mean estimate (≈0.30).
- (B) Bootstrap Distribution Plot: Shows the distribution centered around the mean (≈0.30), confirming robustness.

**Figure S3. Diagnostic plots for the path from Personal Competence to Maintaining Function.**

**A	B**

- (A) Trace Plot: Confirms MCMC convergence with stable stationarity around the mean estimate (≈0.14).
- (B) Bootstrap Distribution Plot: Shows the distribution centered around the mean (≈0.14), with no evidence of bias.

**Figure S4. Diagnostic plots for the path from Personal Competence to Symptom Control.**

**A	B**

- (A) Trace Plot: Confirms MCMC convergence with stable stationarity around the mean estimate (≈0.11).
- (B) Bootstrap Distribution Plot: Shows the distribution centered around the mean (≈0.11), confirming stability.

**Figure S5. Diagnostic plots for the path from Self-Determination to Maintaining Function.**

**A	B**

- (A) Trace Plot: Confirms MCMC convergence with stable stationarity around the mean estimate (≈0.30).
- (B) Bootstrap Distribution Plot: Shows the distribution centered around the mean (≈0.30), indicating robust estimation.

**Figure S6. Diagnostic plots for the path from Emotional Self-Regulation to Symptom Control.**

**A	B**

- (A) Trace Plot: Confirms MCMC convergence with stable stationarity around the mean estimate (≈0.07).
- (B) Bootstrap Distribution Plot: Shows the distribution centered around the mean (≈0.07), confirming estimate stability.

**Figure S7. Diagnostic plots for the path from Symptom Control to Medication Adherence.**

**A	B**

- (A) Trace Plot: Confirms MCMC convergence with stable stationarity around the mean estimate (≈0.06).
- (B) Bootstrap Distribution Plot: Shows the distribution centered around the mean (≈0.06), with no systematic bias.

**Figure S8. Diagnostic plots for the path from Maintaining Function to Medication Adherence.**

**A	B**

- (A) Trace Plot: Confirms MCMC convergence with stable stationarity around the mean estimate (≈0.10).
- (B) Bootstrap Distribution Plot: Shows the distribution centered around the mean (≈0.10), confirming the robustness of the estimate.

**Overall Model Fit Assessment**

The overall model fit was evaluated using the Posterior Predictive p-value (PPP) and the Deviance Information Criterion (DIC). The PPP value was 0.44, which is close to 0.5 and indicates an excellent fit of the model to the data. The effective number of parameters (pD) was estimated at 23.69, and the DIC for the final model was 52.44. These indices, together with the trace plots (confirming convergence) and bootstrap distribution plots (confirming stability), collectively support the adequacy, convergence, and robustness of the specified path model.

References

Gelman, A., & Rubin, D. B. (1992). Inference from iterative simulation using multiple sequences. Statistical Science, 7(4), 457–472. <https://doi.org/10.1214/ss/1177011136>
