# Supplementary material for: Cardiac Self‐Efficacy Mediates the Relationship Between Patient Empowerment and Medication Adherence: A Path Analysis in Patients With Coronary Artery Disease ‐ Empirical Research Quantitative
Source: Nurs Open. 2026 Oct 4;13(10):e70837. doi: 10.1002/nop2.70837 (PMC13635616; doi:10.1002/nop2.70837)
Supplement: Supplementary file 2 [file NOP2-13-e70837-s002.docx]

**Supplementary File 2.** Bayesian Path Analysis as a Sensitivity Analysis

Sensitivity Analysis Using Bayesian Estimation

To evaluate the robustness of the path analysis results considering the ordinal and mixed-response structure of the Medication Adherence Scale (MMAS-8), the hypothesized mediation model was re-estimated using Bayesian estimation in IBM SPSS AMOS 28. Bayesian estimation was implemented using the Markov Chain Monte Carlo (MCMC) algorithm.

The Bayesian analysis produced parameter estimates that were highly consistent with those obtained from the primary Maximum Likelihood (ML) model. The direction, magnitude, and substantive interpretation of all structural paths remained unchanged, supporting the robustness of the primary findings. Therefore, for consistency and ease of interpretation, the ML results are presented in the main manuscript, whereas the Bayesian estimates are reported in this supplementary appendix.

Model fit was evaluated using the Posterior Predictive p-value (PPP). The model demonstrated good fit (PPP = 0.44), which falls within the recommended range of 0.10–0.90, indicating satisfactory agreement between the proposed model and the observed data.

**Table S2.** Bayesian Parameter Estimates for the Path Analysis Mode

|  | Mean | S.E. | S.D. | C.S. |
| --- | --- | --- | --- | --- |
| **Regression weights** |  |  |  |  |
| CSES_symptom.control<--Empowerment_Self.determination | 0.150 | 0.000 | 0.042 | 1.000 |
| CSES_maintain.fuction<--Empowerment_Emotional.self.regulation | 0.304 | 0.000 | 0.119 | 1.000 |
| CSES_maintain.fuction<--Empowerment_Personal.competence | 0.142 | 0.000 | 0.121 | 1.000 |
| CSES_symptom.control<--Empowerment_Personal.competence | 0.108 | 0.000 | 0.091 | 1.000 |
| CSES_maintain.fuction<--Empowerment_Self.determination | 0.298 | 0.000 | 0.056 | 1.000 |
| CSES_symptom.control<--Empowerment_Emotional.self.regulation | 0.066 | 0.000 | 0.089 | 1.000 |
| MA_TOTAL<--CSES_symptom.control | 0.063 | 0.000 | 0.031 | 1.000 |
| MA_TOTAL<--CSES_maintain.fuction | 0.095 | 0.000 | 0.021 | 1.000 |
| **Means** |  |  |  |  |
| Empowerment_Self.determination | 46.320 | 0.001 | 0.504 | 1.000 |
| Empowerment_Emotional.self.regulation | 28.715 | 0.001 | 0.276 | 1.000 |
| Empowerment_Personal.competence | 22.714 | 0.001 | 0.245 | 1.000 |
| **Intercepts** |  |  |  |  |
| CSES_symptom.control | -0.978 | 0.007 | 2.059 | 1.000 |
| MA_TOTAL | 3.358 | 0.001 | 0.306 | 1.000 |
| CSES_maintain.fuction | -7.865 | 0.010 | 2.772 | 1.000 |
| **Covariances** |  |  |  |  |
| Empowerment_Self.determination<->Empowerment_Emotional.self.regulation | 20.553 | 0.011 | 2.644 | 1.000 |
| Empowerment_Emotional.self.regulation<->Empowerment_Personal.competence | 11.014 | 0.005 | 1.322 | 1.000 |
| Empowerment_Self.determination<->Empowerment_Personal.competence | 13.058 | 0.007 | 2.205 | 1.000 |
| e1<->e2 | 17.104 | 0.007 | 2.058 | 1.000 |
| **Variances** |  |  |  |  |
| Empowerment_Self.determination | 67.616 | 0.017 | 5.980 | 1.000 |
| Empowerment_Emotional.self.regulation | 20.314 | 0.008 | 1.798 | 1.000 |
| Empowerment_Personal.competence | 15.965 | 0.005 | 1.412 | 1.000 |
| e1 | 20.952 | 0.005 | 1.854 | 1.000 |
| e2 | 37.784 | 0.012 | 3.345 | 1.000 |
| e3 | 3.187 | 0.001 | 0.281 | 1.000 |

Mean = posterior mean estimate; S.E. = posterior standard error; S.D. = posterior standard deviation; C.S. = critical significance (posterior probability). MA_TOTAL = total score of the 8-item Morisky Medication Adherence Scale (MMAS-8); CSES = Cardiac Self-Efficacy Scale.
